# Supplementary material for: Comparative Transcriptomics for Genes Related to Berberine and Berbamine Biosynthesis in Berberidaceae
Source: Plants (Basel). 2022 Oct 11;11(20):2676. doi: 10.3390/plants11202676 (PMC9610958; doi:10.3390/plants11202676)
Supplement: Supplementary file 1 [file plants-11-02676-s001.zip › Supple Figures in berberis.pptx]

## Slide 1
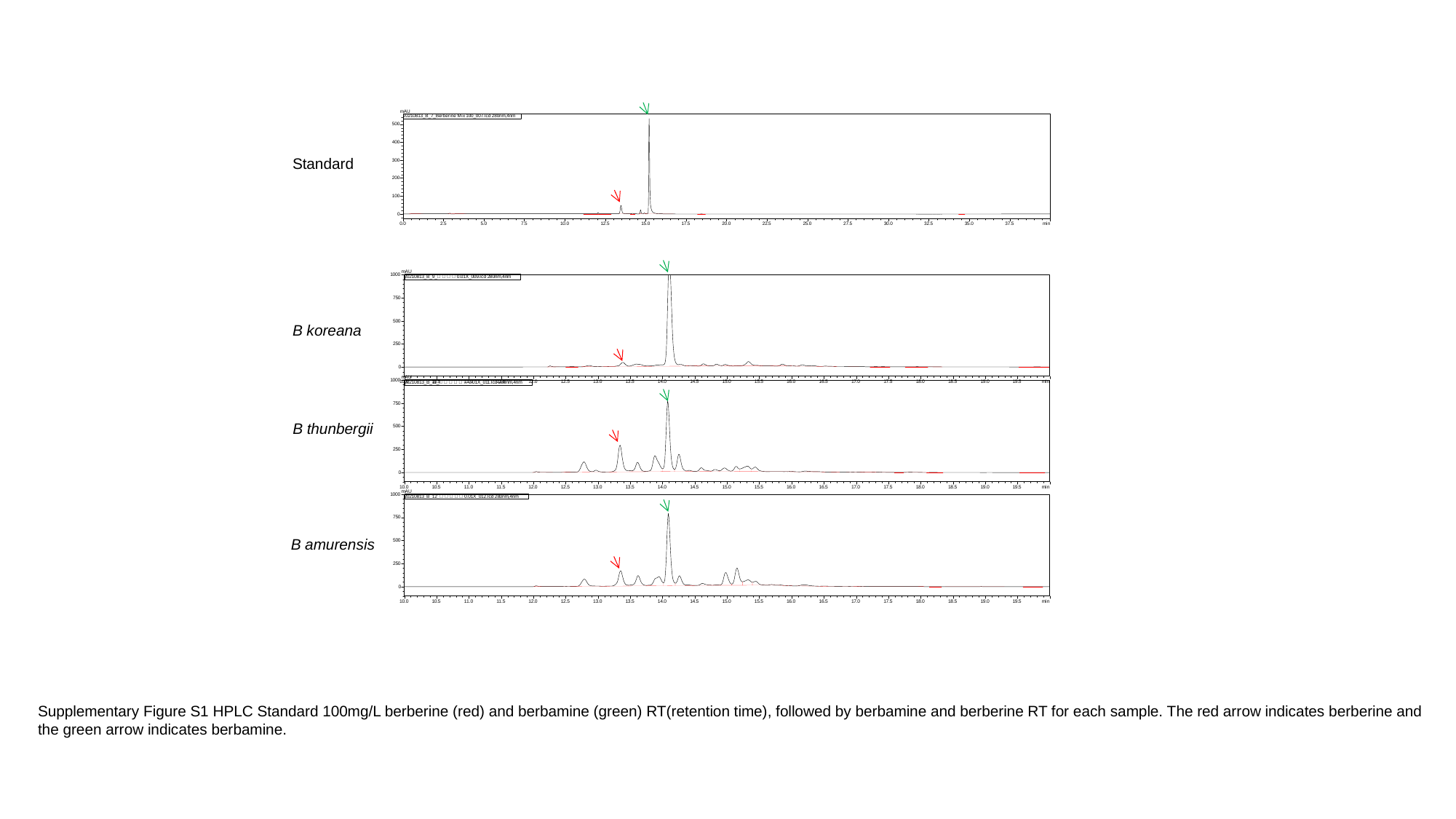

Standard
B koreana
B thunbergii
B amurensis
Supplementary Figure S1 HPLC Standard 100mg/L berberine (red) and berbamine (green) RT(retention time), followed by berbamine and berberine RT for each sample. The red arrow indicates berberine and the green arrow indicates berbamine.

## Slide 2
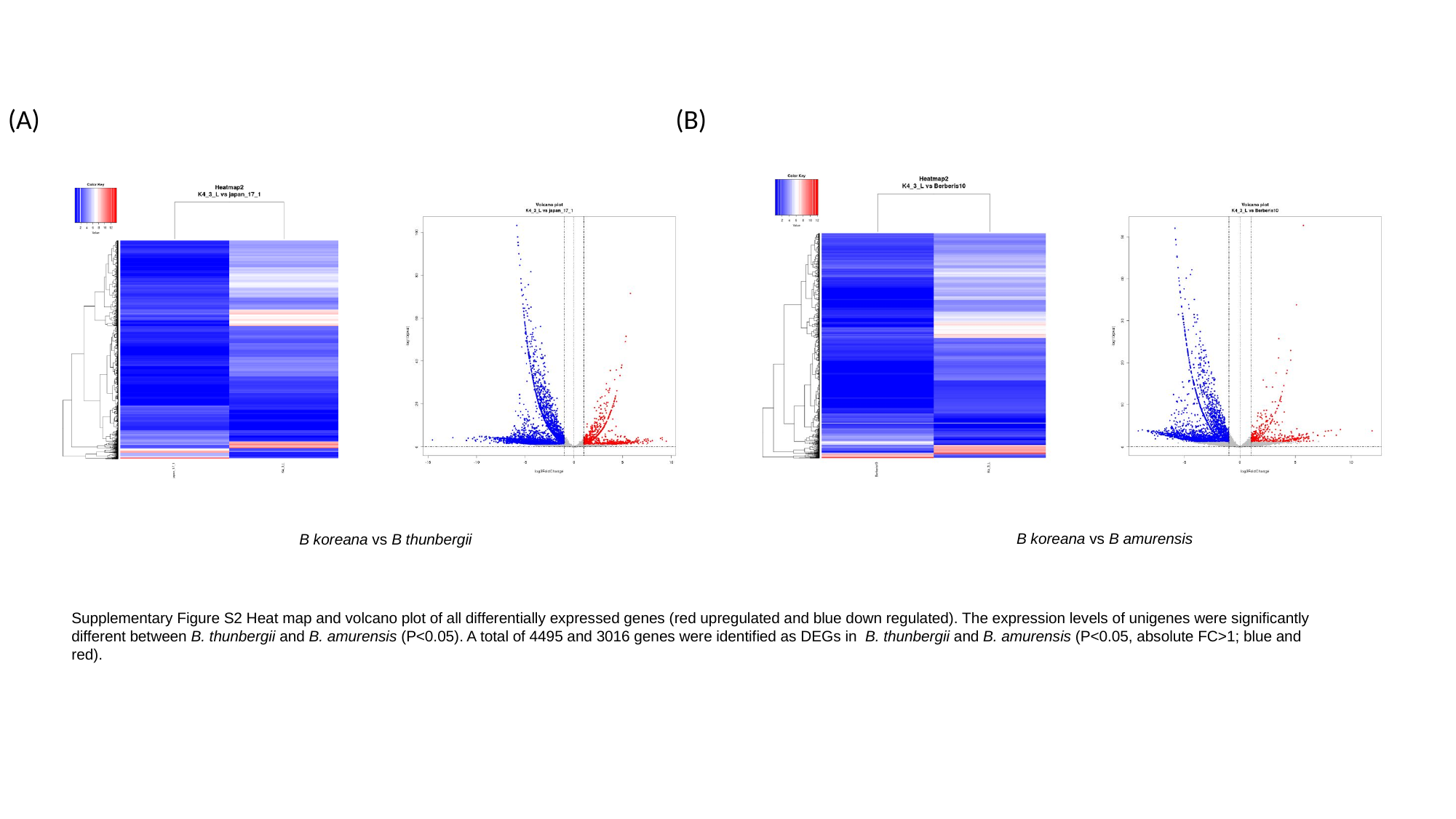

(A)
(B)
B koreana vs B amurensis
B koreana vs B thunbergii
Supplementary Figure S2 Heat map and volcano plot of all differentially expressed genes (red upregulated and blue down regulated). The expression levels of unigenes were significantly different between B. thunbergii and B. amurensis (P<0.05). A total of 4495 and 3016 genes were identified as DEGs in B. thunbergii and B. amurensis (P<0.05, absolute FC>1; blue and red).

## Slide 3
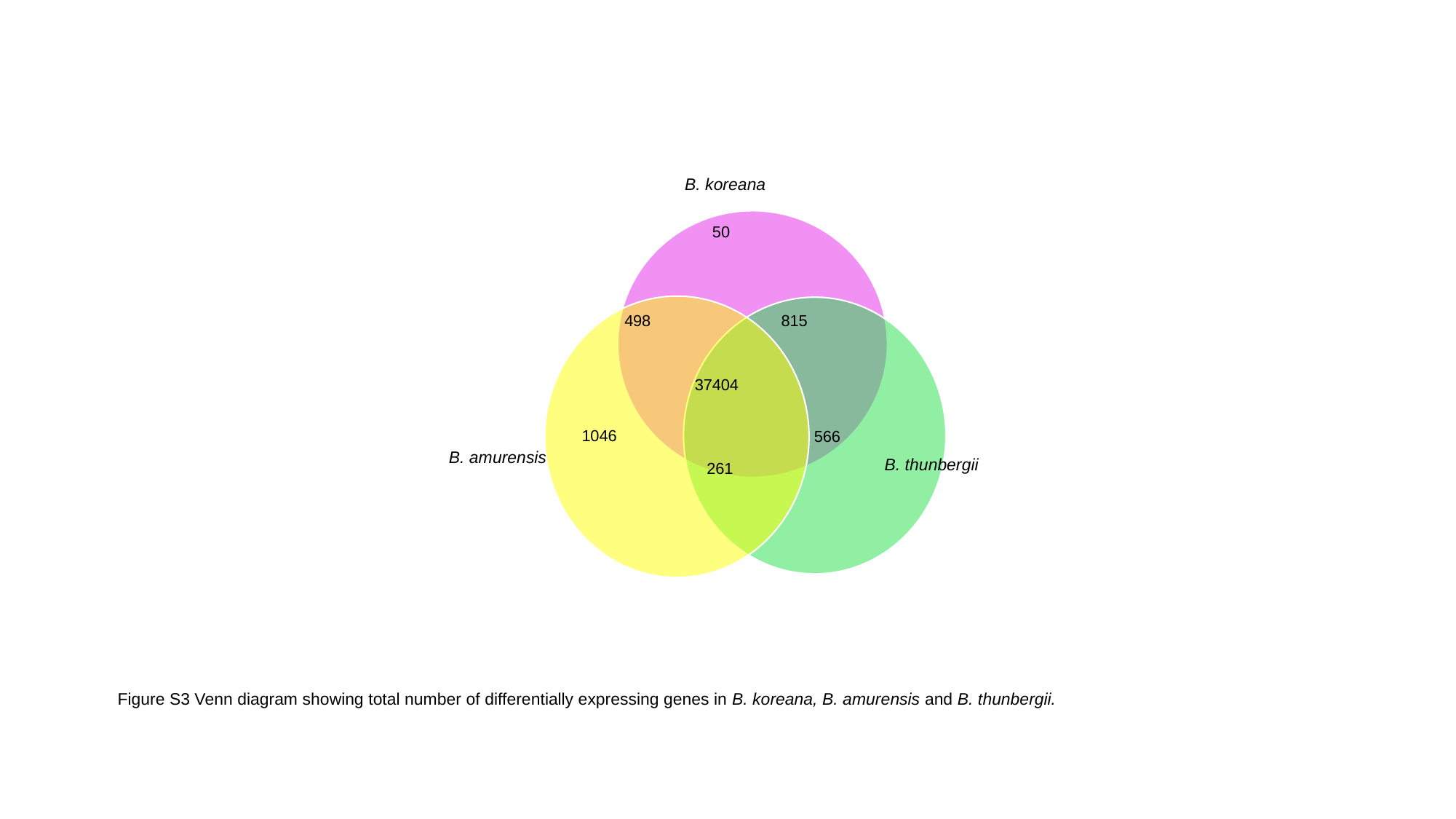

B. koreana
37404
B. amurensis
B. thunbergii
50
498
815
1046
566
261
Figure S3 Venn diagram showing total number of differentially expressing genes in B. koreana, B. amurensis and B. thunbergii.

## Slide 4
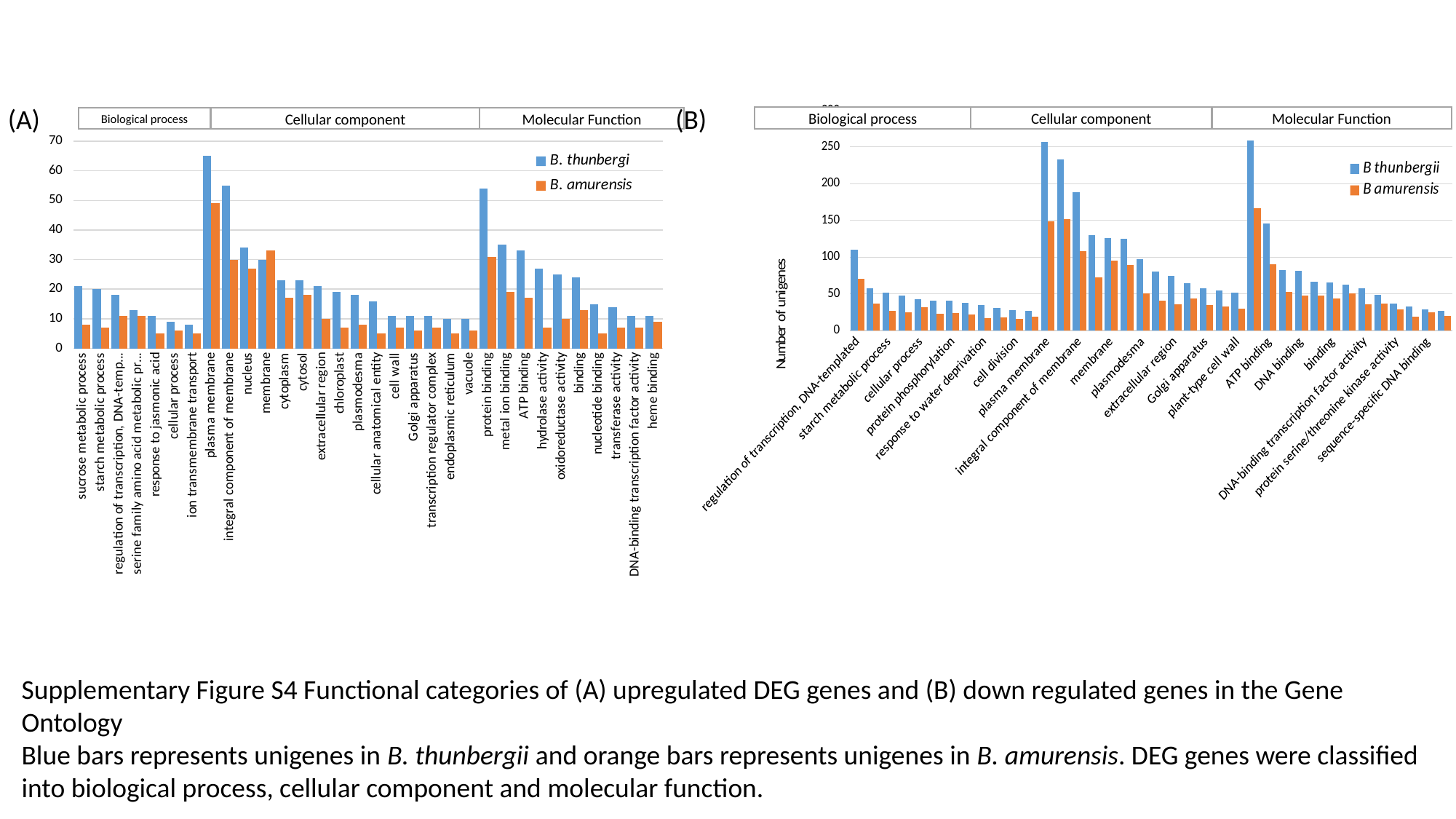

### Chart
| Category | B. thunbergi | B. amurensis |
|---|---|---|
| sucrose metabolic process | 21.0 | 8.0 |
| starch metabolic process | 20.0 | 7.0 |
| regulation of transcription, DNA-templated | 18.0 | 11.0 |
| serine family amino acid metabolic process | 13.0 | 11.0 |
| response to jasmonic acid | 11.0 | 5.0 |
| cellular process | 9.0 | 6.0 |
| ion transmembrane transport | 8.0 | 5.0 |
| plasma membrane | 65.0 | 49.0 |
| integral component of membrane | 55.0 | 30.0 |
| nucleus | 34.0 | 27.0 |
| membrane | 30.0 | 33.0 |
| cytoplasm | 23.0 | 17.0 |
| cytosol | 23.0 | 18.0 |
| extracellular region | 21.0 | 10.0 |
| chloroplast | 19.0 | 7.0 |
| plasmodesma | 18.0 | 8.0 |
| cellular anatomical entity | 16.0 | 5.0 |
| cell wall | 11.0 | 7.0 |
| Golgi apparatus | 11.0 | 6.0 |
| transcription regulator complex | 11.0 | 7.0 |
| endoplasmic reticulum | 10.0 | 5.0 |
| vacuole | 10.0 | 6.0 |
| protein binding | 54.0 | 31.0 |
| metal ion binding | 35.0 | 19.0 |
| ATP binding | 33.0 | 17.0 |
| hydrolase activity | 27.0 | 7.0 |
| oxidoreductase activity | 25.0 | 10.0 |
| binding | 24.0 | 13.0 |
| nucleotide binding | 15.0 | 5.0 |
| transferase activity | 14.0 | 7.0 |
| DNA-binding transcription factor activity | 11.0 | 7.0 |
| heme binding | 11.0 | 9.0 |
### Chart
| Category | B thunbergii | B amurensis |
|---|---|---|
| regulation of transcription, DNA-templated | 110.0 | 71.0 |
| serine family amino acid metabolic process | 58.0 | 37.0 |
| starch metabolic process | 52.0 | 27.0 |
| sucrose metabolic process | 48.0 | 25.0 |
| cellular process | 43.0 | 32.0 |
| obsolete acyl-carrier-protein biosynthetic process | 41.0 | 23.0 |
| protein phosphorylation | 41.0 | 24.0 |
| response to salt stress | 38.0 | 22.0 |
| response to water deprivation | 35.0 | 17.0 |
| phosphorylation | 31.0 | 18.0 |
| cell division | 28.0 | 16.0 |
| protein ubiquitination | 27.0 | 19.0 |
| plasma membrane | 257.0 | 149.0 |
| nucleus | 233.0 | 152.0 |
| integral component of membrane | 188.0 | 108.0 |
| cytosol | 130.0 | 72.0 |
| membrane | 126.0 | 95.0 |
| cytoplasm | 125.0 | 89.0 |
| plasmodesma | 97.0 | 51.0 |
| chloroplast | 80.0 | 41.0 |
| extracellular region | 74.0 | 36.0 |
| transcription regulator complex | 65.0 | 44.0 |
| Golgi apparatus | 58.0 | 35.0 |
| endoplasmic reticulum | 55.0 | 33.0 |
| plant-type cell wall | 52.0 | 30.0 |
| protein binding | 259.0 | 167.0 |
| ATP binding | 146.0 | 90.0 |
| metal ion binding | 82.0 | 53.0 |
| DNA binding | 81.0 | 48.0 |
| nucleotide binding | 67.0 | 48.0 |
| binding | 66.0 | 44.0 |
| hydrolase activity | 63.0 | 51.0 |
| DNA-binding transcription factor activity | 58.0 | 36.0 |
| transferase activity | 49.0 | 37.0 |
| protein serine/threonine kinase activity | 37.0 | 29.0 |
| protein kinase activity | 33.0 | 19.0 |
| sequence-specific DNA binding | 29.0 | 25.0 |
| oxidoreductase activity | 27.0 | 20.0 |Cellular component
Molecular Function
Biological process
(A)
(B)
Cellular component
Biological process
Molecular Function
Supplementary Figure S4 Functional categories of (A) upregulated DEG genes and (B) down regulated genes in the Gene Ontology
Blue bars represents unigenes in B. thunbergii and orange bars represents unigenes in B. amurensis. DEG genes were classified into biological process, cellular component and molecular function.

## Slide 5
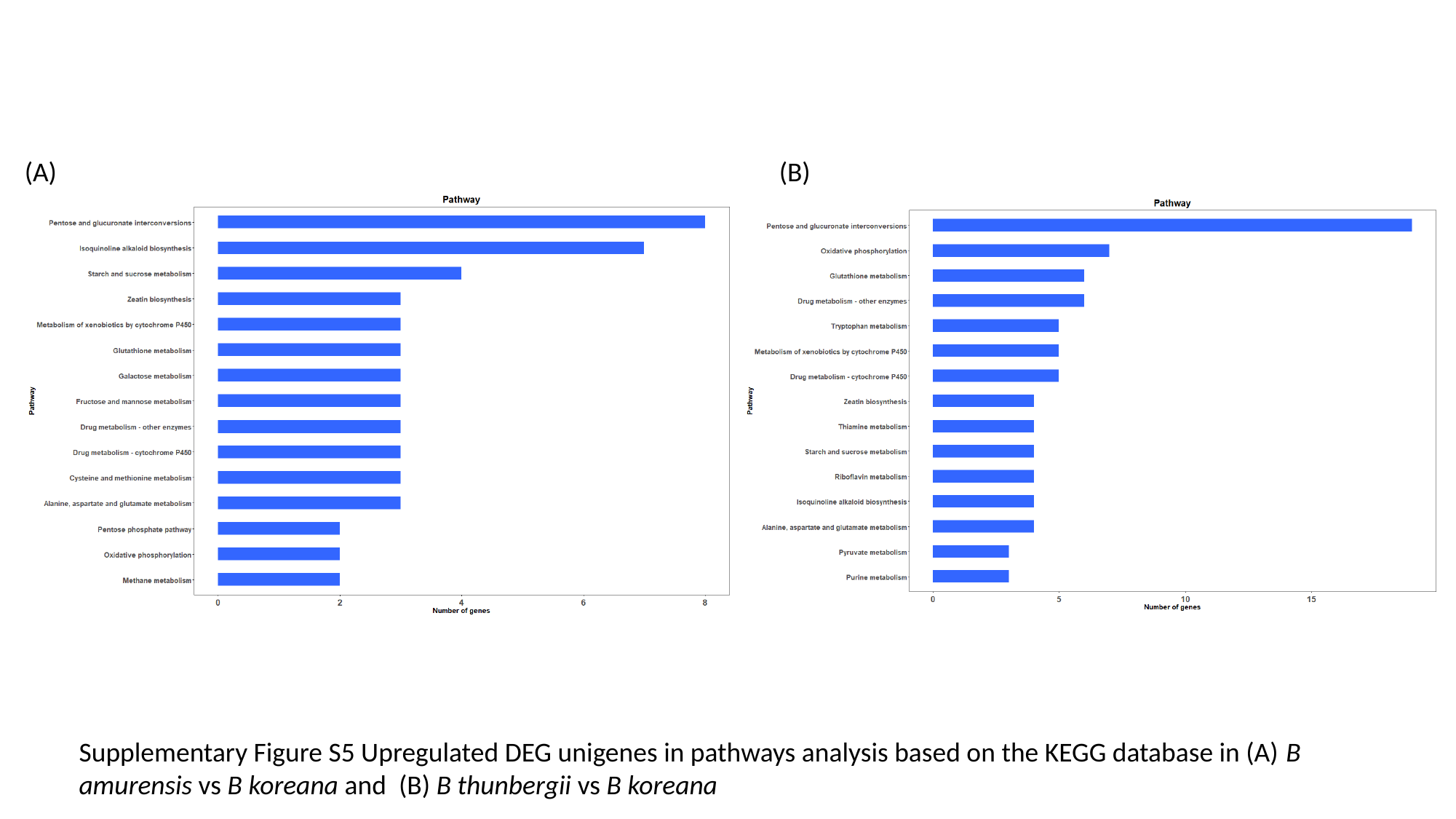

(A)
(B)
Supplementary Figure S5 Upregulated DEG unigenes in pathways analysis based on the KEGG database in (A) B amurensis vs B koreana and (B) B thunbergii vs B koreana

## Slide 6
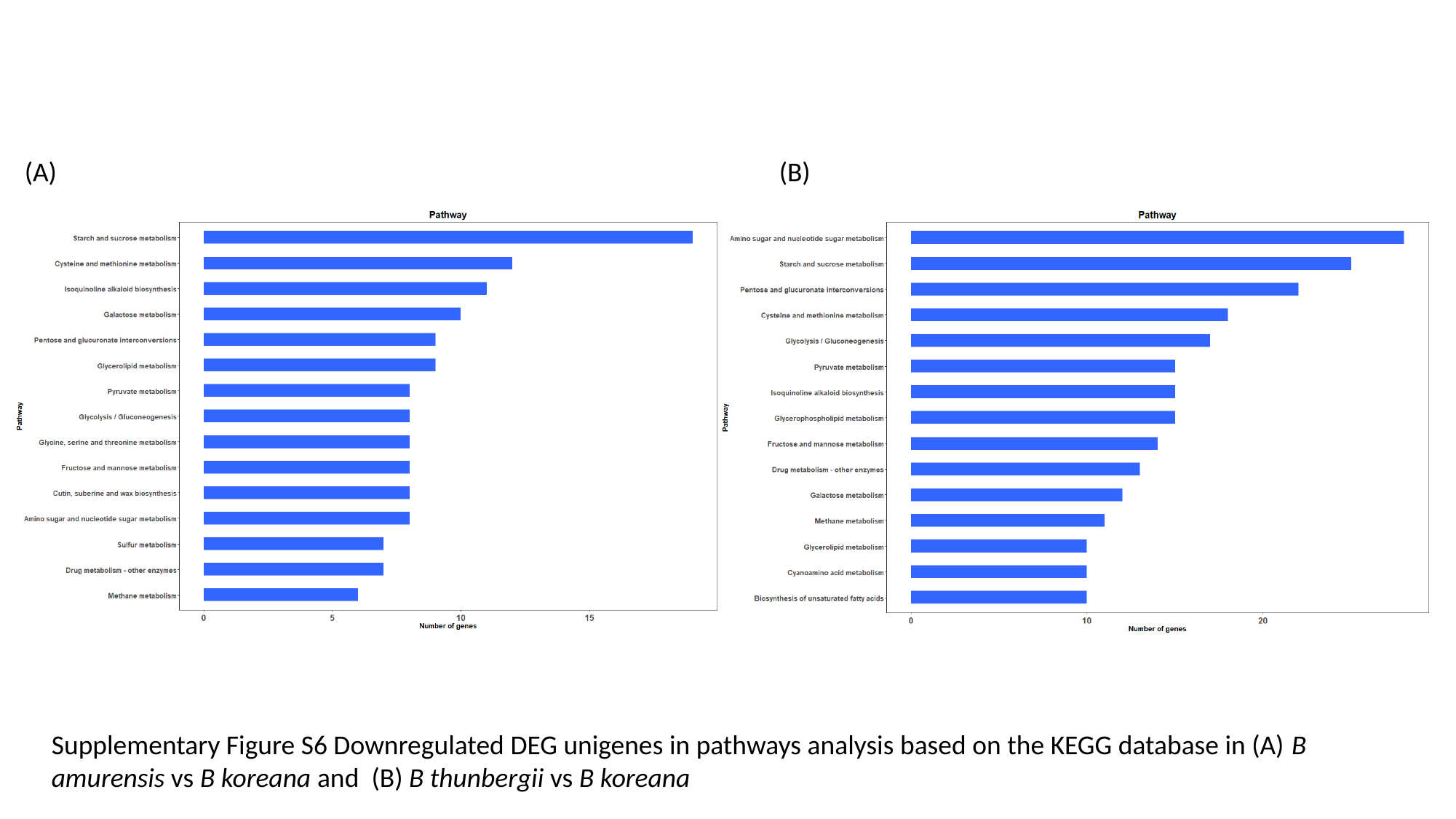

(A)
(B)
Supplementary Figure S6 Downregulated DEG unigenes in pathways analysis based on the KEGG database in (A) B amurensis vs B koreana and (B) B thunbergii vs B koreana
